# physical_validation: A Python package to assess the physical validity of molecular simulation results

**DOI:** 10.21105/joss.03981

**Published:** 2022-01-06

**Authors:** Pascal T. Merz, Wei-Tse Hsu, Matt W. Thompson, Simon Boothroyd, Chris C. Walker, Michael R. Shirts

**Affiliations:** 1 Department of Chemical and Biological Engineering, University of Colorado Boulder, Boulder, CO 80309, United States of America; 2 Boothroyd Scientific Consulting Ltd., 71-75 Shelton Street, London, United Kingdom

## Abstract

Molecular simulations such as molecular dynamics (MD) and Monte Carlo (MC) simulations are powerful tools allowing the prediction of experimental observables in the study of systems such as proteins, membranes, and polymeric materials. The quality of predictions based on molecular simulations depend on the validity of the underlying physical assumptions. physical_validation allows users of molecular simulation programs to perform simple yet powerful tests of physical validity on their systems and setups. It can also be used by molecular simulation package developers to run representative test systems during development, increasing code correctness. The theoretical foundation of the physical validation tests were established by Merz & Shirts (2018), in which the physical_validation package was first mentioned.

## Statement of need

For most of the history of molecular simulation-based research in chemistry, biophysics, physics and engineering, most users of molecular simulation packages were experts that contributed to the code bases themselves or were very familiar with the methodology used. Increased popularity of molecular simulation methods has led to a significantly increased user base and to an explosion of available methods. The simulation packages are faster and more powerful than ever, and even more than before require expertise to avoid using combinations of methods and parameters that could violate physical assumptions or affect reproducibility. Unphysical artifacts have frequently been reported to significantly affect physical observables such as the folding of proteins or DNA, the properties of lipid bilayers, the dynamics of peptides and polymers, or properties of simple liquids (see Merz & Shirts (2018) for further references).

## Functionality

physical_validation tackles the problem of robustness in molecular simulations at two levels. The first level is the end-user level. physical_validation allows users to test their simulation results for a number of deviations from physical assumptions such as the distribution of the kinetic energy, the equipartition of kinetic energy throughout the system, the sampling of the correct ensemble in the configurational quantities, and the precision of the integrator. The combination of these tests allow to cover a wide range of potential unphysical simulation conditions (Merz & Shirts, 2018). This increases the confidence that users can have in their simulation results independently of and in addition to any code correctness tests provided by the developers of their simulation package. These validation tools explain their assumptions and conclusions using comprehensive output and figures, making their use suitable also for users new to the field of molecular simulations. Since physical_validation also returns its conclusions in machine-readable form, it can be included in pipelines allowing results to be tested for physical validity without user interaction. The second level of usage is by code developers. Unphysical behavior might not only result from poor or incompatible parameters and models, it might also stem from coding errors in the simulation programs. physical_validation can therefore be used to regularly run representative simulations as end-to-end tests in a continuous integration setup, ensuring that code changes do not introduce bugs that lead to unphysical results. GROMACS (Abraham et al., 2015), one of the leading MD packages, has been using physical_validation since 2019 to test every release version with a comprehensive set of simulations covering all major code paths.

## Relation to prior work

Shirts (2013) and Merz & Shirts (2018) laid the theoretical foundation for the physical_validation package. Shirts (2013) introduced the ensemble validation tests, and implemented them in a simple python script which was made available accompanied by some examples on github.com/shirtsgroup/checkensemble. This script was used as a base for the ensemble validation tests in physical_validation. Merz & Shirts (2018) built upon the previous work by showing that combining the ensemble tests with kinetic energy distribution and equipartition checks as well as integrator convergence tests could detect many types of unphysical simulation conditions. Merz & Shirts (2018) first mentioned physical_validation and its use in the validation of GROMACS releases.

In the three years since the publication, the software has matured into a stable release. The ensemble tests now also support *μVT* ensembles, covering the full set of ensembles described by Shirts (2013). The user interface, the screen output and the plotting functionality were polished based on user feedback. The API was improved and is now considered stable, and the package can be installed using conda, both of which were much requested features from users looking to use the package in pipelines automating simulation protocols. While the version published in 2018 had no test coverage, the stable release is extensively covered by both unit and regression tests, reaching a test coverage of above 90%. Finally, the documentation was significantly improved based on user feedback.
